# Temporal Lipoma in the Interfascial Fat Pad: A Rare Finding

**DOI:** 10.7759/cureus.98721

**Published:** 2025-12-08

**Authors:** George Tsakotos, George Triantafyllou, Alexandros Samolis, Theodore Troupis, Maria Piagkou

**Affiliations:** 1 Department of Anatomy, School of Medicine, Faculty of Health Sciences, National and Kapodistrian University of Athens, Athens, GRC

**Keywords:** facial nerve, interfascial fat pad, superficial temporal artery, temporal fascia, temporal lipoma

## Abstract

Lipomas are the most frequent benign soft-tissue tumors, but they only infrequently occur in the head and neck region. Among these, those arising in the temporal region, especially from the interfascial fat pad between the two layers of the deep temporal fascia (DTF), are exceptionally rare. This compartment serves as a gliding and protective plane for the overlying temporoparietal fascia and the neurovascular structures coursing within it, including the superficial temporal artery (STA) and the frontal branch of the facial nerve. A routine dissection of an 80-year-old male cadaver revealed a well-encapsulated, lobulated yellowish mass in the right temporal region. The lesion’s fascial boundaries, neurovascular relationships, and anatomical position were carefully documented and compared with established descriptions of temporal fascia anatomy. The mass measured approximately 2.5 × 1.5 cm and was confined between the superficial and deep layers of the DTF, consistent with the temporal (interfascial) fat pad. The frontal branch of the STA coursed superficial to the lesion, while the temporalis muscle lay deep to it, separated by the deep layer of the DTF. The lesion was identified as a lipoma based on its gross appearance and anatomical confinement. This case confirms that the temporal fat pad is a distinct anatomical compartment capable of developing benign lipomatous lesions. Recognition of this relationship is critical during pterional craniotomies, temporal reconstruction, and aesthetic contouring procedures to avoid neurovascular injury, maintain fascial integrity, and preserve temporal contour.

## Introduction

Lipomas are common benign soft-tissue tumors, accounting for 16-50% of all benign mesenchymal neoplasms, yet only about 13% arise in the head and neck region [1]. They typically occur in middle-aged or elderly individuals, show slow progressive enlargement, and are slightly more frequent in males [2].

Lipomas of the temporal region are exceptionally uncommon, especially those originating within the interfascial fat pad, a discrete adipose compartment located between the superficial and deep layers of the deep temporal fascia (DTF) [3,4]. Campiglio et al. [5] first described this compartment as a gliding and cushioning layer that facilitates mastication and facial movement while protecting key neurovascular structures, including the superficial temporal artery (STA) and the frontal branch of the facial nerve (FN), both of which course within the temporoparietal fascia (TPF) [6].

Above the zygomatic arch, the DTF divides into superficial and deep laminae, enclosing the temporal interfascial fat pad. The TPF continues superiorly as the galea aponeurotica and inferiorly as the superficial musculoaponeurotic system (SMAS), forming part of the multilayered facial soft-tissue arrangement described by Cotofana and Lachman [7]. This layered organization underscores the functional importance of the temporal fat pad within the fascial system. Within or just beneath the TPF, the frontal branches of the STA and FN run, making the temporal region particularly vulnerable during surgical intervention [5,8].

Preservation of these fascial planes during procedures such as pterional craniotomy, temporal reconstruction, and aesthetic facial surgery is essential to prevent contour deformities, temporal hollowing, and iatrogenic neurovascular injury [6,8]. A detailed understanding of the fascial layers and their associated fat compartments is therefore critical for neurosurgeons, plastic surgeons, and maxillofacial surgeons.

Clinically, temporal lipomas may remain asymptomatic or present as subtle lateral fullness. Owing to their deep position, they may be misinterpreted as parotid, zygomatic, or temporomandibular pathology unless imaging is performed. Computed tomography (CT) and magnetic resonance imaging (MRI) typically demonstrate a well-circumscribed, homogeneous fatty mass consistent with a lipoma. Given the intimate relationship of the STA and FN with this compartment, surgical excision requires meticulous dissection to avoid neurovascular injury.

The present report describes a right-sided temporal lipoma situated beneath the frontal branch of the STA and confined to the interfascial fat pad. This case contributes to the limited published literature and reinforces the importance of recognizing the temporal fat pad as a distinct anatomical and surgically relevant compartment.

## Case presentation

**Figure 1:**
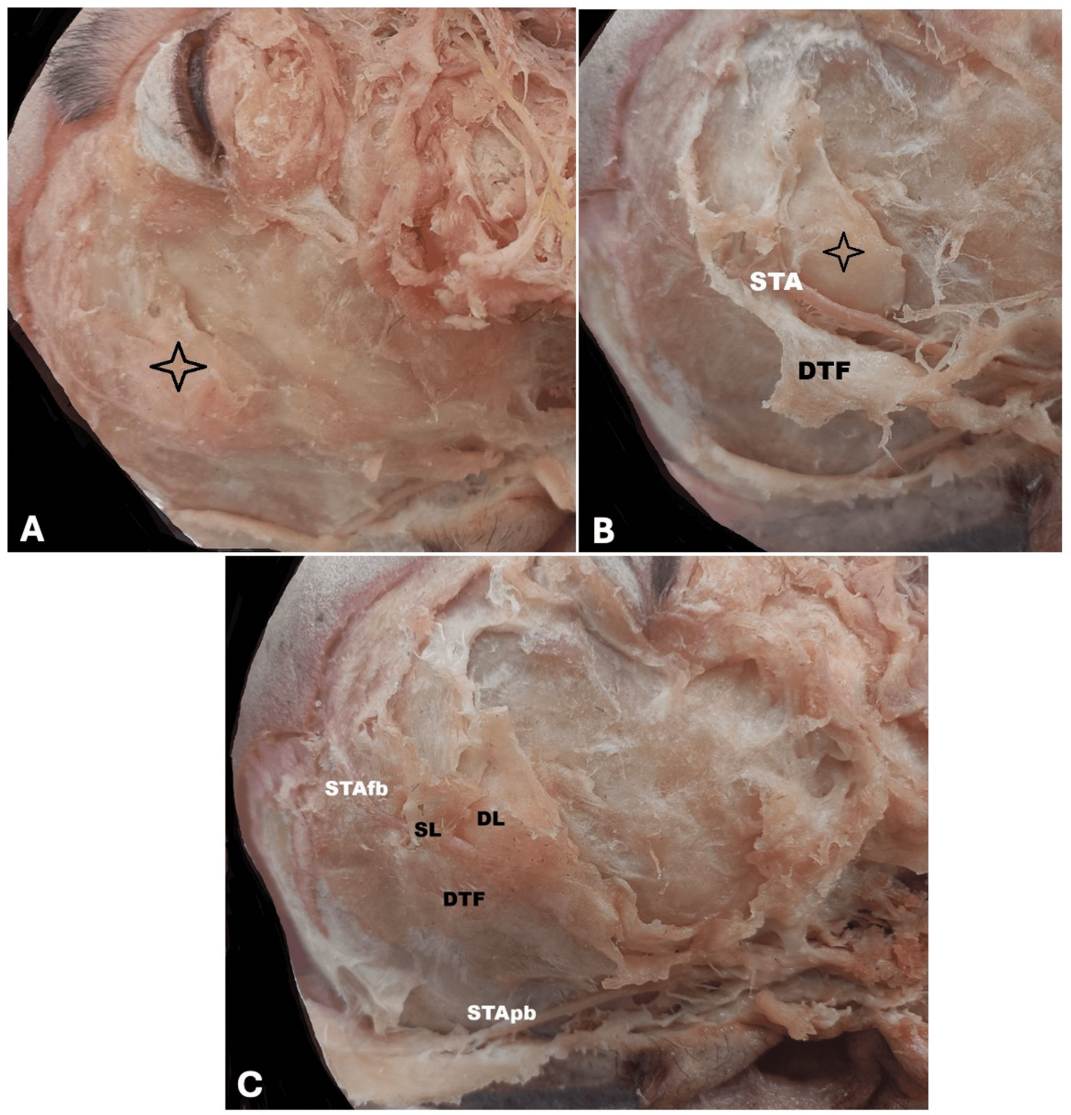
Right temporal region showing the interfascial fat pad. (A) This image demonstrates the temporal (interfascial) fat pad situated between the superficial and deep layers of the deep temporal fascia (DTF). The frontal branch of the superficial temporal artery (STA) is visible coursing superficially over the adipose tissue (asterisk). The deep temporal fascia (DTF) is clearly exposed inferiorly, delineating the deep fascial boundary of the compartment. The lipomatous mass lies directly beneath the STA frontal branch within the interfascial plane. (B) This closer view highlights the superficial layer (SL) and deep layer (DL) of the DTF as they separate superior to the zygomatic arch. The frontal branch of the STA (STAfb) is shown running superficial to the SL of the DTF, while the parietal branch of the STA (STApb) is identified inferiorly. The temporal (interfascial) fat pad lies between the SL and DL. These landmarks confirm the classical description of the interfascial compartment. (C) This panoramic view reveals the entire right hemiface dissection with extensive exposure of the terminal branches of the facial nerve (FN) and the STA. The interfascial fat pad occupies its characteristic position between the two laminae of the DTF. The lipoma sits deep to the STA frontal branch and superficial to the temporalis fascia, confirming its confinement within the interfascial compartment.

During the routine dissection of an 80-year-old male cadaver, a well-circumscribed, lobulated, yellowish mass was observed in the right temporal region. The lesion was situated beneath the frontal branch of the STA and superficial to the temporalis muscle. Meticulous dissection revealed that the mass was enclosed between the superficial and deep layers of the DTF, corresponding to the temporal (interfascial) fat pad. The lesion measured approximately 2.5 × 1.5 cm and had a smooth, encapsulated surface, typical of a benign lipoma. The frontal branch of the STA coursed superficial to the lipoma, within or just beneath the TPF. The temporalis muscle remained intact, separated from the lesion by the deep layer of the DTF. No evidence of infiltration, hemorrhage, or connection with the parotid gland or zygomatic arch was observed. Based on its anatomical position and gross features, the lesion was identified as a lipoma arising from the temporal fat pad, rather than a subcutaneous or intramuscular lipoma (Figure 1).

## Discussion

Lipomas of the temporal region are exceedingly uncommon, particularly those arising from the interfascial fat pad between the layers of the DTF [3,4]. Davies et al. [3] highlighted this rarity, reporting only two comparable cases. The present cadaveric finding expands this limited evidence and confirms that lipomas may originate within this anatomically distinct compartment.

The temporal (interfascial) fat pad, described by Campiglio et al. [5], lies between the superficial and deep layers of the DTF, which diverge superiorly above the zygomatic arch. This adipose layer acts as a cushion and gliding plane, facilitating the smooth motion of the TPF over the temporalis muscle during mastication and facial expression. It also serves as a protective buffer for the neurovascular structures traversing this region, notably the frontal branches of the STA and FN [6,8]. Cotofana and Lachman [7] further demonstrated that the facial soft tissues are organized into layered superficial and deep fat compartments, each with distinct biomechanical functions, reinforcing the notion that the temporal fat pad constitutes an anatomical unit with defined boundaries.

In the present case, the lipoma was located beneath the frontal branch of the STA and superficial to the temporalis muscle, entirely confined to the interfascial fat pad. These findings correlate with previous anatomical studies demonstrating that both the STA and FN lie within or immediately deep to the TPF [5,8]. The mass was well encapsulated, without invasion of surrounding tissue, consistent with a benign lipomatous tumor [1].

Clinically, temporal lipomas often present as slow-growing, painless swellings that are easily mistaken for parotid or zygomatic lesions due to their deep location [2,4]. In addition, postoperative or procedural lipomatous proliferations have been reported, such as the multiple parosteal lipomas documented by Nagano et al. [9], highlighting the potential for adipose tissue response following aesthetic interventions. Imaging remains essential for diagnosis, with CT and MRI showing characteristic features of lipomatous tissue, namely a homogeneous, low-attenuation lesion on CT and hyperintensity on T1-weighted MRI [10].

Surgical management demands awareness of the layered fascial anatomy and the course of the STA and FN. The interfascial approach proposed by Baucher et al. [8] for pterional craniotomy allows safe access to this plane while preserving neurovascular structures. Similarly, during aesthetic procedures, such as fat grafting or temporal hollowing correction, this same anatomical plane can be used to restore contour without compromising FN integrity [6]. Krug et al. [11] reported paradoxical temporal enlargement following interfascial dissection, demonstrating hypertrophy of the superficial temporal fat pad and further supporting the biological responsiveness of temporal adipose tissue. Recognition of the potential for lipomatous lesions in this compartment is therefore essential for surgical planning and differential diagnosis. Dalal et al. [12] emphasize that lipomatous tumors exhibit a wide range of biological behavior, and deeper lesions must always be distinguished from well-differentiated liposarcomas.

The pathogenesis of temporal lipomas is uncertain, but proposed mechanisms include post-traumatic adipocyte proliferation, embryonic fat cell rests, and metabolic influences [1]. Copcu and Sivrioglu [13] demonstrated that trauma may induce lipoma formation either through mechanical herniation of adipocytes or trauma-induced differentiation of preadipocytes triggered by inflammation. The constant movement and vascularity of the temporal fat pad may predispose it to minor repetitive trauma and subsequent adipocytic hyperplasia.

This case enhances understanding of the temporal fascial system and provides direct anatomical evidence of lipoma development within the interfascial fat pad. It also underscores the importance of precise dissection in this area to preserve function and maintain the aesthetic contour of the temporal fossa, key goals in both neurosurgical and reconstructive surgery.

The principal limitation of this report is its cadaveric nature, which precludes correlation with clinical symptoms, imaging findings, and postoperative outcomes. Unlike clinical cases, nerve function and cosmetic outcomes could not be assessed. The absence of pre-dissection CT or MRI also limits direct radiological comparison. The study’s strengths include its detailed dissection, precise documentation of fascial and neurovascular relationships, and confirmation of the spatial relationship of the lipoma to the STA and temporalis fascia. These findings meaningfully complement existing clinical literature and strengthen anatomical understanding of this rare tumor location. The case serves as a valuable anatomical reference for surgeons operating in this intricate region.

## Conclusions

This report describes a rare temporal lipoma located beneath the frontal branch of the STA and confined within the interfascial fat pad between the layers of the DTF. Its encapsulated nature and precise position support the concept of the temporal fat pad as a distinct anatomical compartment capable of developing benign lipomatous lesions. For neurosurgeons and craniofacial surgeons, comprehensive knowledge of the temporal fascial layers and the relationships between the STA and FN is essential to avoid iatrogenic injury, postoperative deformity, and diagnostic error. By integrating this cadaveric observation with contemporary anatomical and clinical literature, the case strengthens understanding of temporal fat-pad pathology. It reinforces the value of the interfascial approach as a safe and anatomically sound surgical corridor.
